# Vibrational energies of some diatomic molecules for a modified and deformed potential

**DOI:** 10.1038/s41598-021-01998-6

**Published:** 2021-11-18

**Authors:** C. A. Onate, I. B. Okon, M. C. Onyeaju, O. Ebomwonyi

**Affiliations:** 1 Physics Programme, Department of Physical Sciences, Landmark University, Omu-Aran, Nigeria; 2 Theoretical Physics Group, Department of Physics, University of Uyo, Uyo, Nigeria; 3 Theoretical Physics Group, Department of Physics, University of Port Harcourt, P.M.B. 5323 Choba, Port Harcourt, Nigeria; 4 Department of Physics, University of Benin, Benin City, Nigeria; 5 Landmark University SDG 4 (Quality Education), Omu-Aran, Nigeria

**Keywords:** Physics, Quantum physics

## Abstract

A molecular potential model is proposed and the solutions of the radial Schrӧdinger equation in the presence of the proposed potential is obtained. The energy equation and its corresponding radial wave function are calculated using the powerful parametric Nikiforov–Uvarov method. The energies of cesium dimer for different quantum states were numerically obtained for both negative and positive values of the deformed and adjustable parameters. The results for sodium dimer and lithium dimer were calculated numerically using their respective spectroscopic parameters. The calculated values for the three molecules are in excellent agreement with the observed values. Finally, we calculated different expectation values and examined the effects of the deformed and adjustable parameters on the expectation values.

## Introduction

In the recent time, exponential-type potential has been the subject of interest in the quantum mechanics which greatly popularized the relativistic and non-relativistic wave equations such as the Schrӧdinger equation, Klein–Gordon equation, Dirac equation and others^1–15^. The approximate solutions of these wave equations have been obtained mostly for one-dimensional system with various exponential-type potentials using different approximation methods developed by different authors. The frequently used methods are Nikiforov–Uvarov method^16,17^, asymptotic iteration method^18^, supersymmetric approach^19,20^, factorization method^21^, exact and proper quantization rule^22,23^. Recently, Ikot et al.^24,25^ have used a new approach called NU Functional analysis method. The different methods have different approach for the solutions of the wave equations but give results that are approximately the same. For instance, the solutions of the radial Schrӧdinger equation under the Deng-Fan potential model has been studied by Dong and Gu^26^ using factorization method. Zhang et al.^27^ and Onate et al.^28^ respectively, also studied the potential via supersymmetry quantum mechanics and parametric Nikiforov–Uvarov method. The results of these authors agreed with one another.

The solutions of the wave equations studied for different potentials, have been applied to the study of several systems such as Theoretic quantities^29–32^ and Thermal properties (mean energy, heat capacity, free energy and entropy)^33–38^. In ref.^27^, the result was used to study the rotation transition frequency for HF. In ref.^28^, the wave function was used to study some theoretic quantities such Shannon entropy and Rényi entropy. In ref.^39^, the problem of $so(2,2)$ was studied under the Pӧschl-Teller potential. Several authors have also studied the energy eigenvalues for many diatomic molecules on molecular dynamics and spectroscopy in the field of chemistry and molecular physics^40,41^. This provides explanations about the dynamics and physical properties of some molecules. The potential energy function involved are used to study the bonding between atoms, hence the predictions to the behaviour of some class of molecules^42^. Some of these potentials can be used to describe some experimental values. Generally, a good empirical internuclear potential function should reproduce the experimental potential energy curves as determined by the RKR method. Considering this, the present study wants to examine an approximate solutions of the Schrӧdinger equation with a new modified and deformed exponential-type molecular potential model confined on a cesium dimer, sodium dimer and lithium dimer. The study also aims to investigate the potential with two different values for each of the deformed parameter and adjustable parameter under the same cesium dimer. This potential has not been reported for any study yet to the best of our understanding.

The cesium dimer is an important molecule that has many applications, e.g. vibrational cooling of molecules, population dynamics, and even coherent control^43–47^. The cesium molecule is an attractive system for examining a possible variation of the electron-to-proton mass ratio and of the fine-structure constant^48^. It is noted that $3^{3} \sum_{g}^{ + }$ state of cesium dimer has a strong Fermi contact interaction with the nuclei, and possesses a large hyperfine splitting^49^. The potential energy curve of the cesium dimer for $3^{3} \sum_{g}^{ + }$ and $a^{3} \sum_{u}^{ + }$ states has been reported in ref.^49,50^. The modified and deformed exponential-type molecular potential model under consideration, is given as

1 $$V(r) = D_{e} - \frac{{D_{e} }}{{Ce^{ - \alpha r} + q_{0} }}\left( {e^{{\alpha r_{e} }} + q_{1} - \frac{{(e^{{\alpha r_{e} }} + q_{1} )^{2} }}{{Ce^{ - \alpha r} + q_{0} }}} \right),$$

where $C$ is a modified parameter, $q_{0}$ is a deformed parameter and $q_{1}$ is an adjustable parameters whose value can be taken as $\pm 1.$ When the value of the adjustable parameter equals the value of the deformed parameter within $\pm 1,$ the results of potential (1) gives other useful results. $D_{e}$ is the dissociation energy $r_{e}$ is the equilibrium bond separation and $\alpha$ is the screening parameter. Its numerical value can be obtain using the formula

2 $$\alpha = \pi c\omega_{e} \sqrt {\frac{2\mu }{{D_{e} }}} + \frac{1}{{r_{e} }}W\left( {\pi c\omega_{e} r_{e} \sqrt {\frac{2\mu }{{D_{e} }}} e^{{ - \pi c\omega_{e} r_{e} \sqrt {\frac{2\mu }{{D_{e} }}} }} } \right),$$

where $W$ is the Lambert function, $\mu$ is the reduced mass, $c$ is the speed of light and $\omega_{e}$ is the vibrational frequency.

## Parametric Nikiforov–Uvarov method

The parametric Nikiforov–Uvarov method is one of the shortest and accurate traditional techniques to solve bound state problems. This method was derived from the conventional Nikiforov–Uvarov method by Tezcan and Sever^17^. According to the authors, the reference equation for the parametric Nikiforov–Uvarov is given as

3 $$\left( {\frac{{d^{2} }}{{ds^{2} }} + \frac{{\alpha_{1} - \alpha_{2} }}{{s(1 - \alpha_{3} s)}}\frac{d}{ds} + \frac{{ - \xi_{1} s^{2} + \xi_{2} s - \xi_{3} }}{{s^{2} (1 - \alpha_{3} s)^{2} }}} \right)\psi (s) = 0.$$

Following the work of these authors, the condition for eigenvalues equation and wave function are respectively given by^17,51^

4 $$n\alpha_{2} - \left( {2n + 1} \right)\alpha_{5} + \alpha_{7} + 2\alpha_{3} \alpha_{8} + n\left( {n - 1} \right)\alpha_{3} + \left( {2n + 1} \right)\sqrt {\alpha_{9} } + \left( {2\sqrt {\alpha_{9} } + \alpha_{3} \left( {2n + 1} \right)} \right)\sqrt {\alpha_{8} } = 0,$$

5 $$\psi_{n,\ell } \left( s \right) = N_{n,\ell } s^{{\alpha_{12} }} \left( {1 - \alpha_{3} s} \right)^{{ - \alpha_{12} - \frac{{\alpha_{13} }}{{\alpha_{3} }}}} \times P_{n}^{{\left( {\alpha_{10} - 1,\frac{{\alpha_{11} }}{{\alpha_{3} }} - \alpha_{10} - 1} \right)}} \left( {1 - 2\alpha_{3} s} \right),$$

The parametric constants in Eqs. (3) and (4) are deduced as follows

6 $$\left. \begin{aligned} & \alpha_{4} = \frac{{1 - \alpha_{1} }}{2},\alpha_{5} = \frac{{\alpha_{2} - 2\alpha_{3} }}{2},\alpha_{6} = \alpha_{5}^{2} + \xi_{1} ,\alpha_{7} = 2\alpha_{4} \alpha_{5} - \xi_{2} ,\alpha_{8} = \alpha_{4}^{2} + \xi_{3} , \\ & \alpha_{9} = \alpha_{3} \left( {\alpha_{7} + \alpha_{3} \alpha_{8} } \right) + \alpha_{6} ,\alpha_{10} = \alpha_{1} + 2\alpha_{4} + 2\sqrt {\alpha_{8} } ,\alpha_{11} = \alpha_{2} - 2\alpha_{5} + 2\left( {\sqrt {\alpha_{9} } + \alpha_{3} \sqrt {\alpha_{8} } } \right), \\ & \alpha_{12} = \alpha_{4} + \sqrt {\alpha_{8} } ,\alpha_{13} = \alpha_{5} - \left( {\sqrt {\alpha_{9} } + \alpha_{3} \sqrt {\alpha_{8} } } \right) \\ \end{aligned} \right\}.$$

## The radial Schrӧdinger equation and the interacting potential

To obtain the energy eigenvalues of the Schrödinger equation with potential (1), we consider the original Schrödinger equation given by

7 $$\left[ { - \frac{{\hbar^{2} }}{2\mu}\left( {\frac{1}{{r^{2} }}\frac{\partial }{\partial r}r^{2} \frac{\partial }{\partial r} + \frac{1}{{r^{2} sin\theta }}\frac{\partial }{\partial \theta }\left( {sin\theta \frac{\partial }{\partial \theta }} \right) + \frac{1}{{r^{2} sin^{2} \theta }}\frac{{\partial^{2} }}{{\partial \phi^{2} }}} \right) + V(r) - E} \right]\psi (r) = 0.$$

Setting the wave function $\psi (r) = U_{n,\ell } (r)Y_{m,\ell } (\theta ,\phi )r^{ - 1} ,$ and consider the radial part of the Schrӧdinger equation, Eq. (7) becomes

8 $$- \frac{{\hbar^{2} }}{2\mu }\frac{{d^{2} U_{n\ell } (r)}}{{dr^{2} }} = E_{n\ell } U_{n\ell } (r) - V(r)U_{n\ell } (r),$$

where $V(r)$ is the interacting potential given in Eq. (1), $E_{n\ell }$ is the non-relativistic energy of the system, $\hbar$ is the reduced Planck’s constant, $\mu$ is the reduced mass, $n$ is the quantum number, $U_{n\ell } (r)$ is the wave function. Substituting Eq. (1) into (8), and by defining $y = \frac{1}{{e^{ - \alpha r} }},$ the radial Schrӧdinger equation with the deformed exponential-type potential turns to be

9 $$\frac{{d^{2} U_{n\ell } (y)}}{{dy^{2} }} + \frac{1 + y}{{y\left( {1 + q_{0} y} \right)}}\frac{{dU_{n\ell } (y)}}{dy} + \frac{{ - Py^{2} + Qy - R}}{{y^{2} \left( {1 + q_{0} y} \right)^{2} }}U_{n\ell } (y) = 0,$$

where

10 $$P = \frac{{2\mu D_{e} }}{{\alpha^{2} \hbar^{2} }}\left( {q_{0}^{2} - \frac{{E_{n,\ell } q_{0}^{2} }}{{D_{e} }} - 2q_{0} (e^{{\alpha r_{e} }} + q_{1} )(1 + e^{{\alpha r_{e} }} + q_{1} )} \right),$$

11 $$Q = \frac{{4\mu D_{e} }}{{\alpha^{2} \hbar^{2} }}\left( {\frac{{E_{n,\ell } q_{0} }}{{D_{e} }} - q_{0} + e^{{\alpha r_{e} }} + q_{1} } \right),$$

12 $$R = \frac{{2\mu (D_{e} - E_{n,\ell } )}}{{\alpha^{2} \hbar^{2} }}.$$

Comparing Eq. (9) with Eq. (3), the parametric constants in Eq. (6) are obtain as follows

13 $$\left. \begin{aligned} & \alpha_{1} = 1,\alpha_{2} = \alpha_{3} = - q_{0} ,\alpha_{4} = 0,\alpha_{5} = \frac{{q_{0} }}{2},\alpha_{6} = \frac{{q_{0}^{2} }}{4} + \frac{{2\mu D_{e} }}{{\alpha^{2} \hbar^{2} }}\left( {q_{0}^{2} - 2q_{0} (e^{{\alpha r_{e} }} + q_{1} ) - \frac{{E_{n,\ell } q_{0}^{2} }}{{D_{e} }} + e^{{\alpha r_{e} }} + q_{1} } \right), \\ & \alpha_{7} = \frac{{4\mu D_{e} }}{{\alpha^{2} \hbar^{2} }}\left( {q_{0} - \frac{{E_{n,} q_{0} }}{{D_{e} }} - e^{{\alpha r_{e} }} - q_{1} } \right),\alpha_{8} = \frac{{2\mu (D_{e} - E_{n,} )}}{{\alpha^{2} \hbar^{2} }},\alpha_{9} = \frac{1}{4}\left( {q_{0}^{2} + \frac{{8\mu D_{e} (e^{{\alpha r_{e} }} + q_{1} )^{2} }}{{\alpha^{2} \hbar^{2} }}} \right), \\ & \alpha_{10} = 1 + 2T_{{V_{1} }} ,\alpha_{11} = - 2q_{0} \left[ {1 + T_{{V_{1} }} } \right] + T_{{V_{2} }} ,\alpha_{12} = T_{{V_{1} }} ,\alpha_{13} = \frac{{q_{0} }}{2} - \frac{1}{2}T_{{V_{2} }} + q_{0} T_{{V_{1} }} ,T_{{V_{1} }} = \sqrt {\frac{{2\mu (D_{e} - E_{n,\ell } )}}{{\alpha^{2} \hbar^{2} }}} , \\ & T_{{V_{2} }} = \sqrt {q_{0}^{2} + \frac{{8\mu D_{e} (e^{{\alpha r_{e} }} + q_{1} )^{2} }}{{\alpha^{2} \hbar^{2} }}} \\ \end{aligned} \right\}.$$

Substituting the parameters in Eq. (13) into Eq. (4), we have the energy equation for the system as

14 $$E_{n} = D_{e} - \frac{{\alpha^{2} \hbar^{2} }}{2\mu }\left[ {\frac{{\frac{{4\mu D_{e} (e^{{\alpha r_{e} }} + q_{1} )}}{{\alpha^{2} \hbar^{2} }} - n(n + 1)q_{0} + \frac{{q_{0} }}{2} - \left( {n + \frac{1}{2}} \right)\sqrt {q_{0}^{2} + \frac{{8\mu D_{e} (e^{{\alpha r_{e} }} + q_{1} )^{2} }}{{\alpha^{2} \hbar^{2} }}} }}{{\sqrt {q_{0}^{2} + \frac{{8\mu D_{e} (e^{{\alpha r_{e} }} + q_{1} )^{2} }}{{\alpha^{2} \hbar^{2} }}} - q_{0} (2n + 1)}}} \right]^{2} ,$$

and the corresponding wave function is obtain when the values of $\alpha_{10}$ to $\alpha_{13}$ in Eq. (6) are substituted into Eq. (5),

15 $$U_{n} (y) = N_{n} y^{{T_{{V_{1} }} }} \left( {1 + q_{0} y} \right)^{{T_{{V_{1} }} \left( {q_{0} - 1} \right) + \frac{1}{2}\left( {T_{{V_{2} }} - q_{0} } \right)}} \left[ {P_{n}^{{\left( {2T_{{V_{1} }} , - \frac{{T_{{V_{2} }} }}{{q_{0} }}} \right)}} \left( {1 + 2q_{0} y} \right)} \right].$$

## Expectation values

In this section, we calculated some expectation values using Hellmann-Faynman Theorey (HFT)^52–56^. When a Hamiltonian $H$ for a given quantum system is a function of some parameter $v,$ the energy-eigenvalue $E_{n}$ and the eigenfunction $U_{n} (v)$ of $H$ are given by

16 $$\frac{{\partial E_{n,} (v)}}{\partial v} = \left\langle {U_{n,} (v)\left| {\frac{\partial H(v)}{{\partial v}}} \right|U_{n,} (v)} \right\rangle ,$$

with the effective Hamiltonian as

17 $$H = - \frac{{\hbar^{2} }}{2\mu }\frac{{d^{2} U_{n\ell } (r)}}{{dr^{2} }} + \frac{{\hbar^{2} }}{2\mu }\frac{\ell (\ell + 1)}{{r^{2} }} + D_{e} - \frac{{D_{e} }}{{e^{ - \alpha r} + q_{0} }}\left( {e^{{\alpha r_{e} }} + q_{1} - \frac{{(e^{{\alpha r_{e} }} + q_{1} )^{2} }}{{e^{ - \alpha r} + q_{0} }}} \right).$$

Setting $v = \mu$ and $v = D_{e,} ,$ we have the expectation values of $p^{2}$ and $V$ respectively are

18 $$\left\langle {p^{2} } \right\rangle_{n} = \left[ \begin{gathered} \left[ {\frac{{4(e^{{\alpha r_{e} }} + q_{1} )^{2} }}{{\lambda_{T} \left( {\lambda_{T} - q_{0} (2n + 1)} \right)}} + \frac{{\alpha^{2} \hbar^{2} }}{2\mu }} \right]\frac{{\left( {A - \left( {n + \frac{1}{2}} \right)\lambda_{T} } \right)^{2} }}{{\mu \left( {\lambda_{T} - q_{0} (2n + 1)} \right)^{2} }} - \hfill \\ \frac{{\alpha^{2} \hbar^{2} \left( {A - \left( {n + \frac{1}{2}} \right)\lambda_{T} } \right)}}{{\mu \left( {\lambda_{T} - q_{0} (2n + 1)} \right)^{2} }}\left( {4D_{e} (e^{{\alpha r_{e} }} + q_{1} )^{2} - \frac{{4D_{e} (e^{{\alpha r_{e} }} + q_{1} )^{2} \left( {n + \frac{1}{2}} \right)}}{{\alpha^{2} \hbar^{2} \lambda_{T} }}} \right) \hfill \\ \end{gathered} \right].$$

19 $$\left\langle V \right\rangle_{n} = 1 + \frac{{4(e^{{\alpha r_{e} }} + q_{1} )^{2} \left( {A\mu - \left( {n + \frac{1}{2}} \right)\lambda_{T} } \right)^{2} }}{{\lambda_{T} \left( {\lambda_{T} - q_{0} (2n + 1)} \right)^{3} }} - A_{T} \left( {\lambda_{T} - (e^{{\alpha r_{e} }} + q_{1} )\left( {n + \frac{1}{2}} \right)} \right)\frac{{\alpha^{2} \hbar^{2} \left( {A\mu - \left( {n + \frac{1}{2}} \right)\lambda_{T} } \right)}}{{\mu \left( {\lambda_{T} - q_{0} (2n + 1)} \right)^{2} }},$$

20 $$\left. \begin{gathered} A = \frac{{4D_{e} (e^{{\alpha r_{e} }} + q_{1} )}}{{\alpha^{2} \hbar^{2} }} + \frac{{q_{0} }}{2} - n(n + 1)q_{0} \hfill \\ \lambda_{T} = \sqrt {q_{0}^{2} + \frac{{8\mu D_{e} (e^{{\alpha r_{e} }} + q_{1} )^{2} }}{{\alpha^{2} \hbar^{2} }}} ,A_{T} = \frac{{4\mu (e^{{\alpha r_{e} }} + q_{1} )}}{{\alpha^{2} \hbar^{2} \lambda_{T} }} \hfill \\ \end{gathered} \right\}.$$

The average deviation of the calculated results from the experimental results is obtained using the formula

21 $$\sigma_{av} = \frac{100}{N}\mathop \Sigma \limits_{n} \left| {\frac{{E_{ER} - E_{CR} }}{{E_{ER} }}} \right|,$$

where $E_{ER}$ is the experimental data, $E_{CR}$ is the calculated values and $N$ is the total number of the experimental data.

## Discussion of result

The comparison of the observed values of RKR and calculated values for $3^{3} \Sigma_{g}^{ + }$ state of cesium dimer with $q_{0} = q_{1} = 1,$ $q_{0} = q_{1} = - 1,$ $D_{e} = 2722.28\;{\text{cm}}^{ - 1} ,$ $r_{e} = 5.3474208$ Å, and $\omega_{e} = 28.891\;{\text{cm}}^{ - 1}$ is reported in Table 1. The results for two values for each of the deformed parameter and adjustable parameter agreed with the observed values of the cesium dimer. However, the results obtained with $q_{0} = q_{1} = - 1$ are higher than their counterpart obtained with $q_{0} = q_{1} = 1.$ In Table 2, the comparison of vibrational energies of sodium dimer and lithium dimer respectively are reported. When the deformed parameter and the adjustable parameter are taken as one with $D_{e} = 79885\;{\text{cm}}^{ - 1} ,$ $r_{e} = 1.097$ Å, $\omega_{e} = 2358.6\;{\text{cm}}^{ - 1} ,$ the results agreed with the observed values of $5^{1} \Delta_{g}$ state of sodium dimer. Taken the deformed parameter and adjustable parameter respectively as minus one, with $D_{e} = 2722.28\;{\text{cm}}^{ - 1} ,$ $r_{e} = 4.173$ Å and $\omega_{e} = 65.130\;{\text{cm}}^{ - 1} ,$ the results obtained correspond to the observed values of lithium dimer.

**Table 1:** Comparison of theoretical values with experimental values for the vibrational energy levels of the modified deformed exponential-type molecular potential for $3^{3} \sum_{g}^{ + }$ state of cesium dimer.

$n$	RKR^1^ cm^−1^	$q_{0} = q_{1} = 1$ cm^−1^	RKR^49^ cm^−1^	$q_{0} = q_{1} = - 1$ cm^−1^
0	14.4248	14.42647874	19,477.5507	19,477.55769
1	43.1680	43.17554991	19,506.2939	19,506.29999
2	71.7657	71.77608344	19,534.8916	19,534.90041
3	100.2211	100.2450879	19,563.3470	19,563.35986
4	128.5375	128.5580953	19,591.6634	19,591.68592
5	156.7182	156.7410068	19,619.8441	19,620.43756
6	184.7663	184.7735524	19,647.8922	19,648.55903
7	212.6851	212.6619860	19,675.8110	19,677.08704
8	240.4778	240.4268832	19,703.6037	19,704.38389
9	268.1477	268.2399412	19,731.2736	19,732.74500
10	295.6980	296.0578830	19,758.8239	19,759.62187
11	323.1320	323.3428999	19,786.2579	19,786.99999
12	350.4529	351.0106935	19,813.5788	19,814.29857

**Table 2:** Comparison of theoretical values with experimental (in cm^−1^) values for the vibrational energy levels of the modified deformed exponential-type molecular potential for $5^{1} \Delta_{g}$ state sodium dimer and $a^{3} \Sigma_{u}^{ + }$ state of lithium dimer.

$n$	$Na_{2}$^57^	$q_{0} = q_{1} = 1$ Present results	$Li_{2}$^57^	$q_{0} = q_{1} = - 1$ Present results
0	60.33300	60.30301255	31.8570	31.76487540
1	180.3730	180.2529821	90.4530	90.32373499
2	299.5550	299.3039824	142.523	142.3750821
3	417.8710	417.2590837	188.240	188.0291646
4	535.3130	534.4058726	227.679	227.3301735
5	651.8720	650.2238934	260.837	260.3399997
6	767.5390	765.0058797	287.665	287.1458022
7	882.3050	879.4527801	308.098	307.8296283
8	996.1620	993.0282669	322.155	322.4300795
9	1109.100	1104.263984	330.170	331.0234210
10	1221.113	1215.346550	333.269	333.6470384

To deduce the effect of the deformed and adjustable parameters on the numerical values and discrepancy of the calculated results from the experimental data, we used the formula given in Eq. (28). For cesium dimer, the average percentage deviation for $q_{0} = q_{1} = 1$ is 0.0038% while the average percentage deviation for $q_{0} = q_{1} = - 1$ is 0.0002%. For sodium dimer with $q_{0} = q_{1} = 1,$ the average percentage deviation is 0.0342% while the average percentage deviation for lithium dimer with $q_{0} = q_{1} = - 1,$ is 0.0016%. In Table 3, we presented the numerical results for the two different expectation values calculated in Eq. (20) and Eq. (21). The effect of the deformed and adjustable parameters on the expectation values can be seen in Table 3. For $\left\langle {p^{2} } \right\rangle ,$ the values obtained with $q_{0} = q_{1} = 1$ are higher than their counterpart obtained with $q_{0} = q_{1} = - 1.$ However, for $\left\langle V \right\rangle_{n}$ the values obtained with $q_{0} = q_{1} = - 1$ are higher than their counterpart obtained with $q_{0} = q_{1} = 1.$

**Table 3:** Expectation values at various quantum states with $$\hbar = \mu = 1,$$$$r_{e} = 0.4,$$$$\alpha = 0.25$$ and $$D_{e} = 5$$.

*n*	$q_{0} = q_{1} = - 1.$		$q_{0} = q_{1} = 1.$	
	$\left\langle {p^{2} } \right\rangle_{n}$	$\left\langle V \right\rangle_{n}$	$\left\langle {p^{2} } \right\rangle_{n}$	$\left\langle V \right\rangle_{n}$
0	− 0.068637482	0.295409752	22.32941128	− 15.96118652
1	− 0.111005221	0.626165798	22.44835649	− 16.66269191
2	− 0.094264753	0.781132946	22.33359455	− 17.40250843
3	− 0.072565924	0.855219469	21.92055901	− 18.17986161
4	− 0.056599797	0.888914740	21.12697090	− 18.99025081
5	− 0.044980410	0.901338383	19.84830239	− 19.82213968
6	− 0.033277583	0.902210621	17.95340272	− 20.65071832
7	− 0.016766628	0.896751247	15.28301985	− 21.42578612
8	0.008774554	0.887879382	11.65940036	− 22.04728521
9	0.046903527	0.877275751	6.930904930	− 22.31342118
10	0.100553144	0.865926536	1.123376051	− 21.80368609

**Figure 1:**
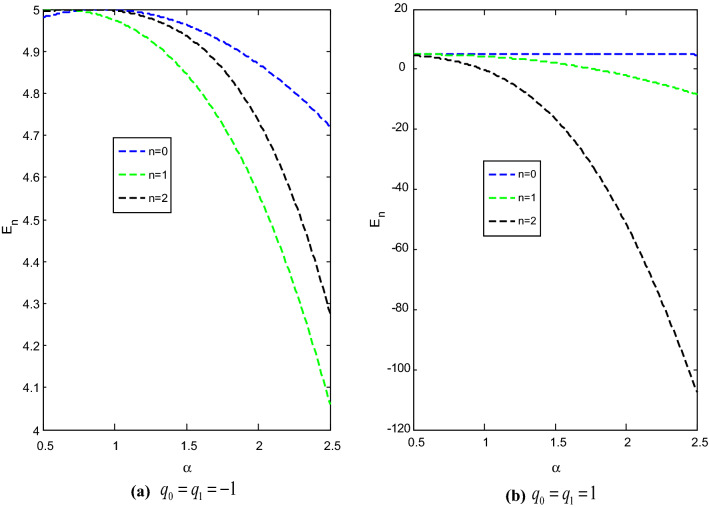
Variation of energy $$E_{n}$$ against the screening parameter $$\alpha ,$$ with $$\hbar = \mu = 1,$$$$r_{e} = 0.4$$ and $$D_{e} = 5$$.

The effect of the screening parameter on the energy eigenvalues with two values each of the deformed parameter and adjustable parameter are shown in Fig. 1. In each case, the energy of the system varies inversely with the screening parameter. The energy of the system for $q_{0} = q_{1} = 1$ are lower than the energy of the system for $q_{0} = q_{1} = - 1.$

## Conclusion

The solutions of a one-dimensional Schrӧdinger equation is obtained for a molecular potential model using parametric Nikiforov–Uvarov method. By changing the numerical values of the deformed parameter and adjustable parameter, the results obtained for different molecules agreed with experimental values. However, the results obtained with $q_{0} = q_{1} = - 1$ are closer to the experimental values compared with the results obtained with $q_{0} = q_{1} = 1.$ The results for lithium dimer are more closer to the experimental values followed by the results for cesium dimer obtained with $q_{0} = q_{1} = - 1.$
